# Supplementary material for: Structural distortions control scaling of exciton binding energies in two-dimensional Ag/Bi double perovskites
Source: Nanoscale. 2025 Sep 29;17(41):23924–9. doi: 10.1039/d5nr03010b (PMC12495387; doi:10.1039/d5nr03010b)
Supplement: NR-017-D5NR03010B-s001 [file NR-017-D5NR03010B-s001.pdf]

Supplementary Information (SI) for Nanoscale.
This journal is © The Royal Society of Chemistry 2025

Supplementary Information for Structural Distortions Control Scaling of Exciton Binding Energies in Two-Dimensional Ag/Bi Double Perovskites

Pierre Lechifflart,* Raisa-Ioana Biega, and Linn Leppert*

MESA+ Institute for Nanotechnology, University of Twente, 7500 AE Enschede, The Netherlands

E-mail: p.lechifflart@utwente.nl; l.leppert@utwente.nl

Computational Methods

We compute the ground-state electronic properties of our systems using Density Functional Theory (DFT), as implemented in the simulation package `QuantumESPRESSO`,[1,2] benefiting from GPU acceleration,[3] within the Generalized Gradient Approximation using the Perdew-Burke-Ernzerhof (PBE) functional.[4] We use norm-conserving fully relativistic pseudopotentials from the PseudoDojo platform,[5] with the following valence electron configurations: Cs $5s^2$ $5p^6$ $6p^1$, Ag $4s^2$ $4p^6$ $4d^{10}$ $5s^1$, Bi $5d^{10}$ $6s^2$ $6p^3$, Br $4s^2$ $4p^5$, C $2s^2 2p^2$, N $2s^2 2p^3$. We use a plane-wave basis set cut-off at an energy of 60 Ry. To sample reciprocal space, we use a $4 \times 4 \times 2$ Monkhorst-Pack grid of $\mathbf{k}$-points, converged to yield accurate electronic densities and total energies. Spin-orbit coupling (SOC) is taken into account self-consistently. To obtain the quasiparticle corrections to the electronic energies, we perform G_0W_0 calculations with the software package `BerkeleyGW`,[6,7] using a spinor implementation to account for spin-orbit coupling.[8] We converged the computational parameters for $\mathbf{2}^{\text{exp}}$, the structure with the largest number of atoms, and used the same parameters for all model systems. We sum over 1200 electronic state to compute the dielectric matrix, with a cutoff of 8 Ry. The electronic Green's function G_0 was calculated with the same number of bands. With these settings, our G_0W_0 band gaps are converged to within 0.1 eV. Convergence of the dielectric matrix and the G_0W_0 gap are reported in Figure S2.

The Bethe-Salpeter equation (BSE) was solved on a $12 \times 12 \times 2$ reciprocal-space grid, using of the interpolation scheme implemented in `BerkeleyGW`. The convergence of these parameters are reported in Table S3. These convergence tests were performed for the $\mathbf{1}^{\text{exp}-\text{Cs}}$ model. 32 valence and 32 conduction bands are used to diagonalize the BSE Hamiltonian for the experimental structures, and 8 valence and 8 conduction bands for the model structures. The fine grids used at the `absorption` step are all shifted by a random vector, as this was proven to speed up the convergence.[9] The peaks in the absorption spectra are broadened with a Gaussian smearing of 50 meV. The $\mathbf{k}$-dependent weight of the transitions that constitute the excitons are checked in post-processing. This allows to identify the origin of the different

excitonic states in the Brillouin Zone.

The exciton wavefunction is a six-dimensional quantity that depends on electron and hole positions. We calculate its spatial extent using a method based on the average electron-hole separation introduced in Ref. 10 and documented in detail in our earlier work.[11] The second moment of the correlation function between the electron and the hole positions is computed over a large supercell of $12 \times 12 \times 2$. The supercell size is chosen such that it contains all of the electron distribution when the hole is fixed at its center. We then subtract the average electron position from the second moment of the correlation function, and this difference defines the exciton extent. We also average over two different hole positions on different apical Br atoms within the inorganic layer and report the distance that contains 95% of the electron distribution.

Distortion parameters of the experimental structures

The experimental structures depicted in Fig. 1 of the main text exhibit octahedral distortions within the inorganic layers. The Metal-Br bond lengths are given in Table S1. In this table, the term equatorial refers to a Metal-Br bond in the plane of the inorganic layers. Apical refers to a Metal-Br bond pointing out of plane. For $\mathbf{2}^{\rm exp}$, we distinguish between the apical bonds that are in between two sublayers, and the apical terminal bond which are pointing at the organic molecules between two adjacent layers. We also display relevant bond angles

Table S1: Bond lengths in Å for different bonds within the experimental structures.

**Bond Type**	$\mathbf{1}^{\rm exp}$	$\mathbf{2}^{\rm exp}$
Ag-Br equatorial	3.00	2.86
Ag-Br apical	-	3.16
Ag-Br apical terminal	2.68	2.67
Bi-Br equatorial	2.84	2.84
Bi-Br apical	-	2.76
Bi-Br apical terminal	2.88	2.96

for $\mathbf{1}^{\rm exp}$ and $\mathbf{2}^{\rm exp}$ in Fig. S1. It is to be noted that the octahedral distortions are much more

pronounced in $\mathbf{2}^{\mathrm{exp}}$ than in $\mathbf{1}^{\mathrm{exp}}$. The octahedral rotations are slightly more pronounced for $\mathbf{1}^{\mathrm{exp}}$, and the octahedral tilting is about the same for the two systems.

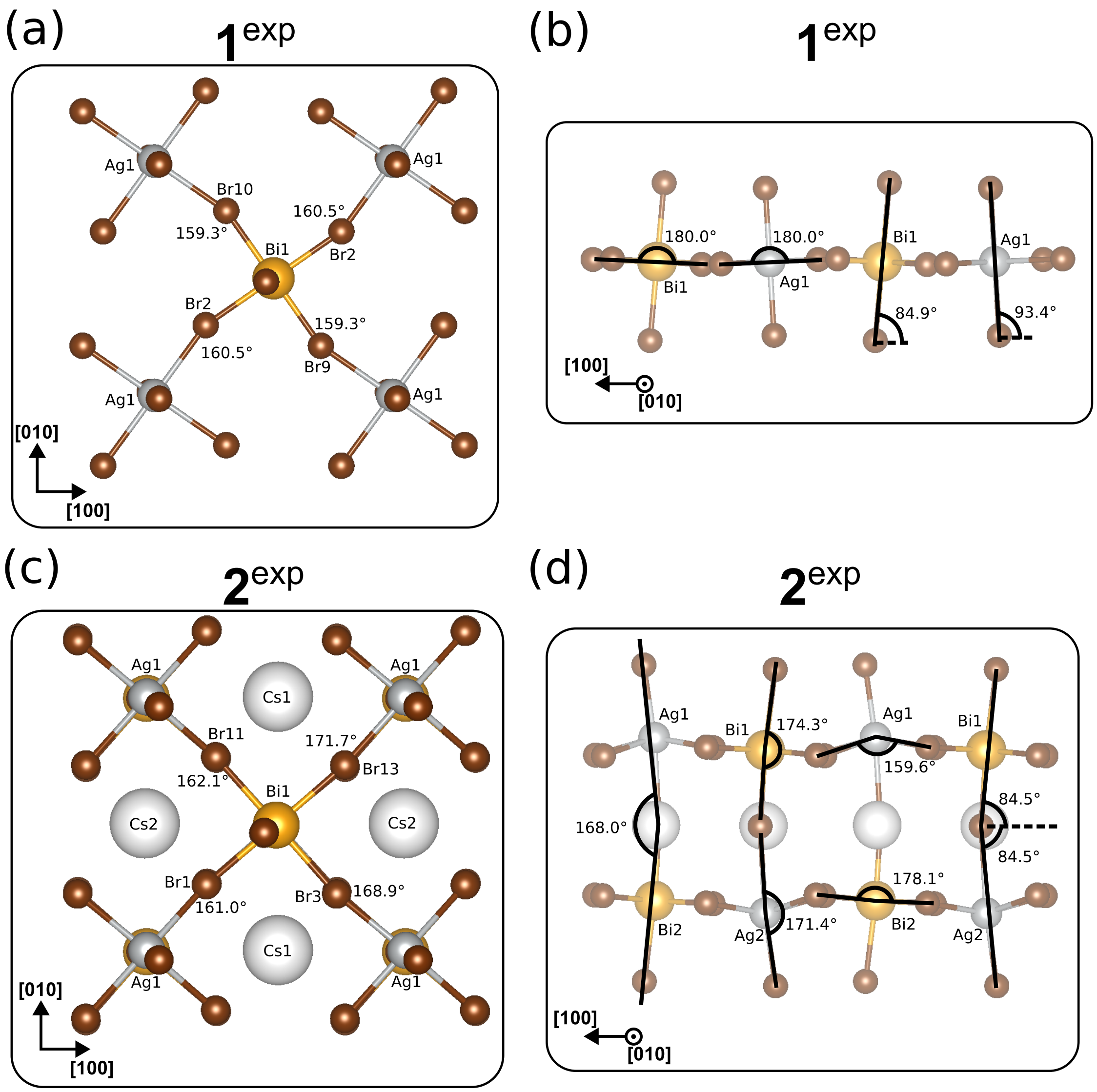

Figure S1: (a) Top view and (b) side view of a layer of $\mathbf{1}^{\mathrm{exp}}$. (c) Top view and (d) side view of a single layer of $\mathbf{2}^{\mathrm{exp}}$.

Construction of Model Systems

To build the model structures, we replace the organic molecules by Cesium atoms. This conserves the charge balance and does not contribute electronic states close to the band-

edges. In the models called $\mathbf{1}^{\mathrm{exp-Cs}}$ and $\mathbf{2}^{\mathrm{exp-Cs}}$, all the other atoms belonging to the inorganic layers are kept at the same positions as in the experimental structures, *i.e.*, the octahedral distortions are preserved. Additionally, we build another pair of model structures, denoted $\mathbf{1}^{\mathrm{model}}$ and $\mathbf{2}^{\mathrm{model}}$, where the organic molecules are replaced by Cs atoms and all octahedral distortions are eliminated. Unless specified otherwise, the model systems are built to have the same interlayer distance d as the experimental structures. We build octahedra with all metal-Br bonds equal to the ones found in the bulk form of $Cs_2AgBiBr_6$, and where all the angles between such bonds are 90 degrees. These structures are depicted in Figure 2c of the main paper. Furthermore, we construct these model systems with different stacking patterns. Keeping two adjacent inorganic layers on top of each other results in the Dion-Jacobson (DJ) stacking pattern, while shifting one layer by half an in-plane diagonal results in the Ruddlesden-Popper (RP) stacking pattern, as illustrated schematically in the insets of Figure 3a-b of the main paper. For adjacent layers in the DJ stacking patterns, Ag and Bi octahedra are alternated in the out-of-plane direction. We tested that this was the most stable configuration for all interlayer distances.

For all model structures, we relax the positions of the Cs atoms, using a BFGS minimization algorithm implemented in `QuantumESPRESSO`, such that the forces acting on them are less than 3×10^{-7} eV/Å.

Optical Absorption Spectra of Model Systems

Optical absorption spectra of $\mathbf{1}^{\mathrm{model}}$ and $\mathbf{2}^{\mathrm{model}}$ are reported in Figure S3. We note that the excitonic peak at about 2.8 eV observed in $\mathbf{2}^{\mathrm{exp}}$ does not appear in $\mathbf{2}^{\mathrm{model}}$. This is because of the lower number of valence and conduction bands used in the solution of the BSE for the model systems, which does not allow for the resolution of excitons above a certain energy threshold. However, this has no influence on the first dark and bright excitons, which are

the central focus of this work. Our absorption spectra are in good agreement with Ref. 12. Some differences can be attributed to the fact that we use one-shot G_0W_0@PBE+SOC which is known to underestimate the band gap of perovskites[13–15] whereas Palummo *et al.* use eigenvalue self-consistent GW. This explains the difference in the onset energy of the spectra, and also the peak separation. The differences in the relative peak intensities likely come from the different sampling of reciprocal space. In this work, a $12 \times 12 \times 2$ grid is used whereas in Ref. 12, a $10 \times 10 \times 2$ is used.

Table S2: Lowest direct band gap as calculated with DFT-PBE and G_0W_0@PBE, static dielectric constant as computed within the random phase approximation, and binding energy of the first dark and first bright excited state.

System	Direct Gap (eV)		ϵ_∞	Exciton binding energy (meV)	
	PBE	G_0W_0@PBE		dark	bright
$\mathbf{1}^{\text{exp}}$	1.64	2.96	3.03	736	654
$\mathbf{1}^{\text{exp}-\text{Cs}}$	1.59	3.02	2.77	834	785
$\mathbf{2}^{\text{exp}}$	2.01	3.10	3.63	893	734
$\mathbf{2}^{\text{exp}-\text{Cs}}$	1.96	3.06	3.33	879	720
$\mathbf{1}^{\text{model}}$	1.67	2.78	3.22	664	608
$\mathbf{2}^{\text{model}}$	1.50	2.77	3.77	592	513
$Cs_2AgBiBr_6$	1.78	2.36	5.19	350	240

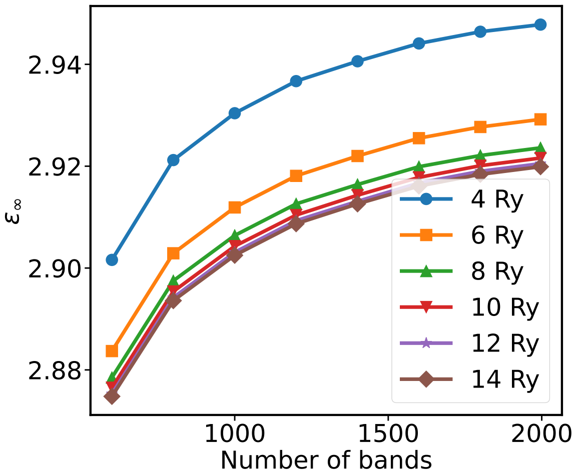

(a) Macroscopic dielectric function

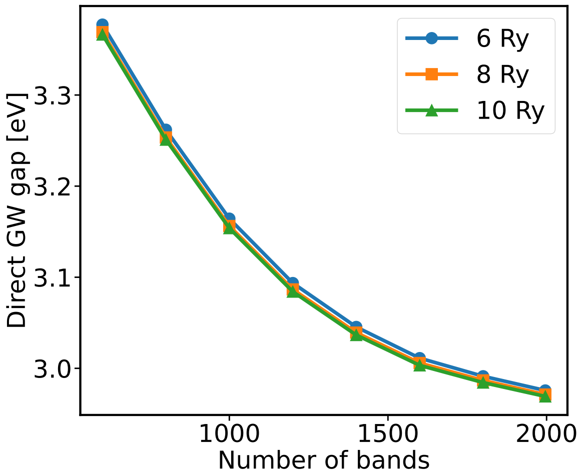

(b) G_0W_0 direct bandgap at Γ

Figure S2: Convergence plots of the dielectric constant and the G_0W_0 bandgap for the $\mathbf{1}^{\mathrm{exp-Cs}}$ model system as a function of the number of bands used in the summation of the dielectric matrix and in the summation of the correlation self-energy, and the dielectric matrix cutoff energy.

Table S3: Convergence of exciton binding energies for different number of bands, coarse and fine grid combinations. Values obtained for the 1^{exp-Cs} model.

coarse grid	ε_∞	fine grid	bands in kernel		bands in absorption		exciton binding energy (eV)		
			v	c	v	c	1st dark	1st bright	2nd bright
2x2x1	2.838	8x8x1	20	20	4	4	1.011	1.118	1.112
		8x8x1	20	20	8	8	1.039	1.013	1.007
		10x10x1					0.989	0.960	0.947
		8x8x2	20	20	8	8	0.988	0.963	0.960
		10x10x2					0.972	0.946	0.930
		12x12x2					0.981	0.955	0.938
4x4x2	2.766	8x8x1	20	20	8	8	0.666	0.623	0.616
		10x10x1					0.730	0.692	0.675
		8x8x2	20	20	8	8	0.781	0.738	0.730
		12x12x2					0.793	0.756	0.750

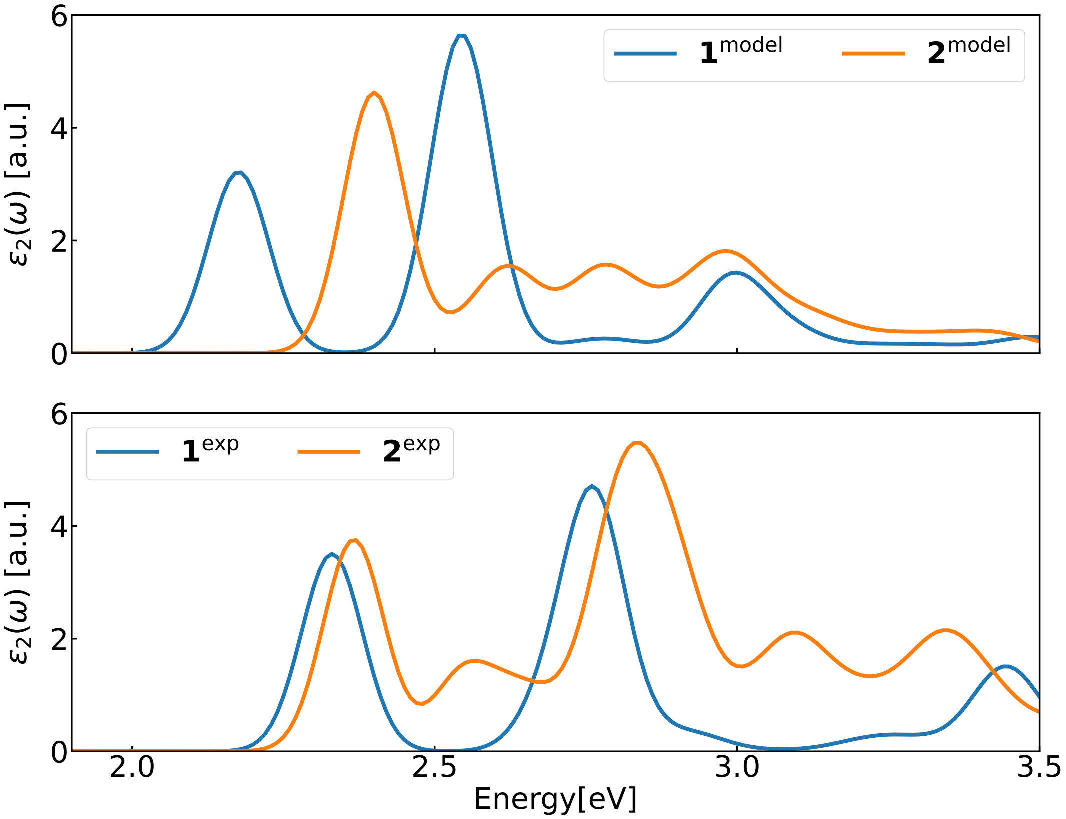

Figure S3: Optical absorption spectra of $\mathbf{1}^{\text{model}}$, $\mathbf{2}^{\text{model}}$ and $\mathbf{1}^{\text{exp}}$, $\mathbf{2}^{\text{exp}}$. Blue lines are for systems with n=1 and orange lines for n=2. The two spectra on the bottom row are the same as those reported in Figure 1.

References

(1) Giannozzi, P.; Baroni, S.; Bonini, N.; Calandra, M.; Car, R.; Cavazzoni, C.; Ceresoli, D.; Chiarotti, G. L.; Cococcioni, M.; Dabo, I. et al. QUANTUM ESPRESSO: a modular and open-source software project for quantum simulations of materials. *Journal of Physics: Condensed Matter* **2009**, *21*, 395502.

(2) Giannozzi, P.; Andreussi, O.; Brumme, T.; Bunau, O.; Nardelli, M. B.; Calandra, M.; Car, R.; Cavazzoni, C.; Ceresoli, D.; Cococcioni, M. et al. Advanced capabilities for materials modelling with Quantum ESPRESSO. *Journal of Physics: Condensed Matter* **2017**, *29*, 465901.

(3) Giannozzi, P.; Baseggio, O.; Bonfà, P.; Brunato, D.; Car, R.; Carnimeo, I.; Cavazzoni, C.; de Gironcoli, S.; Delugas, P.; Ferrari Ruffino, F. et al. Quantum ESPRESSO toward the exascale. *The Journal of Chemical Physics* **2020**, *152*, 154105.

(4) Perdew, J. P.; Burke, K.; Ernzerhof, M. Generalized Gradient Approximation Made Simple. *Phys. Rev. Lett.* **1996**, *77*, 3865–3868.

(5) van Setten, M.; Giantomassi, M.; Bousquet, E.; Verstraete, M.; Hamann, D.; Gonze, X.; Rignanese, G.-M. The PseudoDojo: Training and grading a 85 element optimized norm-conserving pseudopotential table. *Computer Physics Communications* **2018**, *226*, 39–54.

(6) Deslippe, J.; Samsonidze, G.; Strubbe, D. A.; Jain, M.; Cohen, M. L.; Louie, S. G. BerkeleyGW: A Massively Parallel Computer Package for the Calculation of the Quasi-particle and Optical Properties of Materials and Nanostructures. *Comp. Phys. Comm.* **2012**, *183*, 1269–1289.

(7) Del Ben, M.; da Jornada, F. H.; Canning, A.; Wichmann, N.; Raman, K.; Sasanka, R.; Yang, C.; Louie, S. G.; Deslippe, J. Large-Scale GW Calculations on Pre-Exascale HPC Systems. *Comp. Phys. Comm.* **2019**, *235*, 187–195.

(8) Barker, B. A.; Deslippe, J.; Lischner, J.; Jain, M.; Yazyev, O. V.; Strubbe, D. A.; Louie, S. G. Spinor GW/Bethe-Salpeter Calculations in BerkeleyGW: Implementation, Symmetries, Benchmarking, and Performance. *Phys. Rev. B* **2022**, *106*, 115127.

(9) Rohlfing, M.; Louie, S. G. Electron-hole excitations and optical spectra from first principles. *Phys. Rev. B* **2000**, *62*, 4927–4944.

(10) Sharifzadeh, S.; Darancet, P.; Kronik, L.; Neaton, J. B. Low-Energy Charge-Transfer Excitons in Organic Solids from First-Principles: The Case of Pentacene. *J. Phys. Chem. Lett.* **2013**, *4*, 2197.

(11) Biega, R. I.; Filip, M. R.; Leppert, L.; Neaton, J. B. Chemically Localized Resonant Excitons in Silver-Pnictogen Halide Double Perovskites. *J. Phys. Chem. Lett.* **2021**, *12*, 2057–2063.

(12) Palummo, M.; Postorino, S.; Borghesi, C.; Giorgi, G. Strong Out-of-Plane Excitons in 2D Hybrid Halide Double Perovskites. *Appl. Phys. Lett.* **2021**, *119*, 051103.

(13) Giorgi, G.; Yamashita, K.; Palummo, M. Nature of the Electronic and Optical Excitations of Ruddlesden–Popper Hybrid Organic–Inorganic Perovskites: The Role of the Many-Body Interactions. *J. Phys. Chem. Lett.* **2018**, *9*, 5891–5896.

(14) Filip, M. R.; Giustino, F. GW Quasiparticle Band Gap of the Hybrid Organic-Inorganic Perovskite CH3NH3PbI3: Effect of Spin-Orbit Interaction, Semicore Electrons, and Self-Consistency. *Phys. Rev B* **2014**, *90*, 245145.

(15) Leppert, L. Excitons in Metal-Halide Perovskites from First-Principles Many-Body Perturbation Theory. *The Journal of Chemical Physics* **2024**, *160*, 050902.
